# Healthcare resource utilization in patients with pulmonary hypertension associated with chronic obstructive pulmonary disease (PH-COPD): a real-world data analysis

**DOI:** 10.1186/s12890-023-02698-9

**Published:** 2023-11-21

**Authors:** Tracey Weiss, Aimee M. Near, Xiaohui Zhao, Dena Rosen Ramey, Tania Banerji, Handing Xie, Steven D. Nathan

**Affiliations:** 1 Center for Observational and Real-World Evidence, Merck & Co., Inc, 351 N Sumneytown Pike, PA North Wales, 19454 USA; 2 IQVIA, Durham, NC USA; 3 Advanced Lung Disease and Lung Transplant Program, Inova Fairfax Hospital, Falls Church, VA USA

**Keywords:** Chronic obstructive pulmonary disease (COPD), Comorbidities, Healthcare cost, Healthcare resource utilization, Hospitalization, Pulmonary hypertension secondary to COPD

## Abstract

**Rationale:**

There is a lack of real-world characterization of healthcare costs and associated cost drivers in patients with pulmonary hypertension secondary to chronic obstructive pulmonary disease (PH-COPD).

**Objectives:**

To examine (1) excess healthcare resource utilization (HCRU) and associated costs in patients with PH-COPD compared to COPD patients without PH; and (2) patient characteristics that are associated with higher healthcare costs in patients with PH-COPD.

**Methods:**

This study analyzed data from the IQVIA PharMetrics^®^ Plus database (OCT2014-MAY2020). Patients with PH-COPD were identified by a claims-based algorithm based on PH diagnosis (ICD-10-CM: I27.0, I27.2, I27.20, I27.21, I27.23) after COPD diagnosis. Patients aged ≥40 years and with data available ≥12 months before (baseline) and ≥6 months after (follow-up) the first observed PH diagnosis were included. Patients with other non-asthma chronic pulmonary diseases, PH associated with other causes, cancer, left-sided heart failure (HF), PH before the first observed COPD diagnosis, or right-sided/unspecified HF during baseline were excluded. Patients in the PH-COPD cohort were matched 1:1 to COPD patients without PH based on propensity scores derived from baseline patient characteristics. Annualized all-cause and COPD/PH-related (indicated by a primary diagnosis of COPD or PH) HCRU and costs during follow-up were compared between the matched cohorts. Baseline patient characteristics associated with higher total costs were examined in a generalized linear model in the PH-COPD cohort.

**Results:**

A total of 2,224 patients with PH-COPD were identified and matched to COPD patients without PH. Patients with PH-COPD had higher all-cause HCRU and annual healthcare costs ($51,435 vs. $18,412, *p*<0.001) than matched COPD patients without PH. Among patients with PH-COPD, costs were primarily driven by hospitalizations (57%), while COPD/PH-related costs accounted for 13% of all-cause costs. Having a higher comorbidity burden and a prior history of COPD exacerbation were major risk factors for higher total all-cause costs among patients with PH-COPD.

**Conclusions:**

Treatment strategies focusing on preventing hospitalizations and managing comorbidities may help reduce the burden of PH-COPD.

**Supplementary Information:**

The online version contains supplementary material available at 10.1186/s12890-023-02698-9.

## Background

Pulmonary hypertension (PH) can develop as a complication in patients with chronic obstructive pulmonary disease (COPD), particularly among patients with advanced disease [1]. Pre-capillary PH is defined hemodynamically by a mean pulmonary arterial pressure (mPAP) > 20 mmHg accompanied by a pulmonary vascular resistance (PVR) ≥ 3 WU and a pulmonary capillary wedge pressure (PCWP) ≤15 mmHg [2]. PH secondary to COPD (PH-COPD) is a sub-category of Group 3 PH (PH associated with lung disease or hypoxemia) as defined by the World Health Organization [2].

PH-COPD can be difficult to diagnose as the symptoms (e.g. dyspnea) overlap with those of the underlying COPD [3]. In addition, patients may have other etiologies for their PH (e.g., patients with left heart disease [Group 2 PH-LHD] or pulmonary arterial hypertension [Group 1, PAH]) with COPD as a comorbidity [4]. The reported prevalence of PH among patients with COPD varies widely (5–53%) [5], depending on the definition of PH, the COPD population studied, and the method of measuring pulmonary arterial pressure (echocardiography [ECHO] vs. right heart catheterization [RHC]). This may, in part, be attributed to guidelines discouraging formal diagnosis of PH-COPD by RHC for most COPD patients due to the invasiveness of the procedure and lack of approved therapies for those suspected of having PH [6, 7].

The presence of PH-COPD is associated with significant adverse consequences compared to patients with COPD but no PH. Specifically, these patients have more impaired functional capabilities [8], increased frequency of hospitalizations [9], and worse overall survival [10]. Additionally, PH has been associated with higher 30-day hospital re-admission rates, greater hospitalization costs, and increased overall healthcare resource utilization (HCRU) among patients with COPD who experience acute exacerbations [11]. However, there is a lack of real-world data on HCRU and costs of PH-COPD among broader groups of patients with COPD in the US. Thus, this claims-based study sought to estimate the excess burden of PH-COPD on all-cause and disease-related HCRU and associated direct health care costs as well as explore characteristics that are associated with higher total all-cause healthcare costs of PH-COPD.

## Methods

### Study design and patient population

**Fig. 1:**
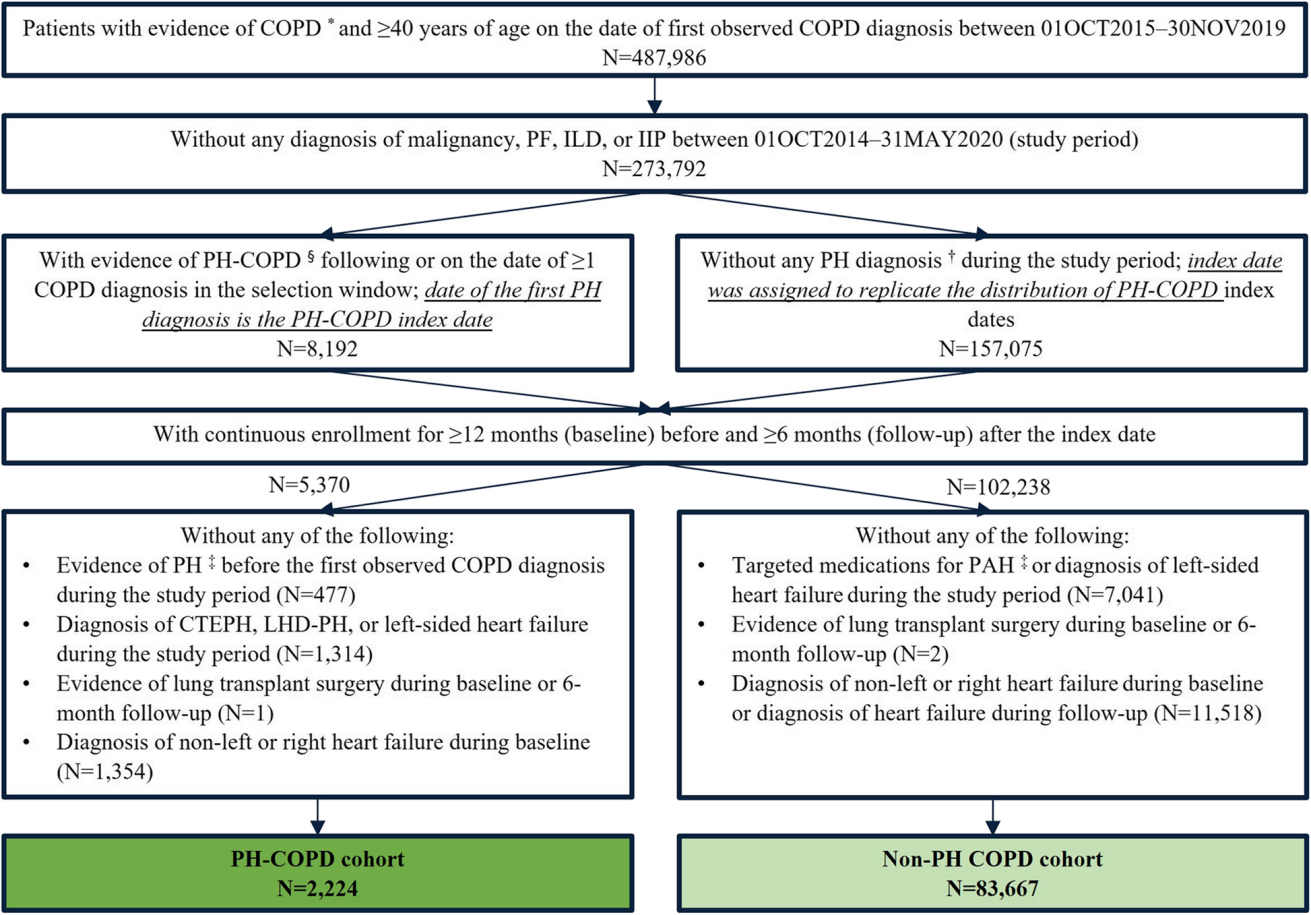
Patient selection. COPD, chronic obstructive pulmonary disease; CTEPH, chronic thromboembolic pulmonary hypertension; Dx, diagnosis; IIP, idiopathic interstitial pneumonia; ILD, interstitial lung disease; LHD-PH, PH associated with left heart disease; PF, pulmonary fibrosis. ^*^ COPD was identified by ≥1 inpatient or ≥2 outpatient diagnoses of PH (ICD-10-CM codes of J40.x, J41.x, J42, J43.x, J44.x) that were ≥30 days apart. ^§^ PH-COPD was identified by ≥1 inpatient or ≥2 outpatient diagnoses of PH (ICD-10-CM codes of I27.0, I27.2, I27.20-23) that were ≥30 days apart. ^†^ PH diagnoses were indicated by ICD-9-CM codes of 416.0 and 416.8 as well as ICD-10-CM codes of I27.0, I27.2x. ^‡^ Evidence of PH included any PH Dx in b and medications approved for PAH (phosphodiesterase-5 inhibitors, guanylate cyclase stimulators, endothelin receptor antagonists, and prostacyclin). 1,688 patients were excluded due to the lack of COPD diagnosis prior to the first PH diagnosis

An observational cohort study was conducted using data from a nationally-representative commercial insurance claims database (IQVIA PharMetrics® Plus) with data extracted for the period from October 1, 2014 to May 31, 2020 (study period). As depicted in Fig. 1, patients with COPD, identified by ≥ 1 inpatient or ≥ 2 outpatient diagnoses that were ≥ 30 days apart, who were ≥ 40 years old on the date of the first observed COPD diagnosis were identified between October 1, 2015 and November 30, 2019 (selection window). Included patients with COPD were categorized into two cohorts. A PH-COPD cohort was identified by a claims-based algorithm, based on ≥ 1 inpatient or ≥ 2 outpatient diagnoses for PH that were ≥ 30 days apart, with ≥ 1 COPD diagnosis preceding the first observed PH diagnosis (index date). Patients were required to have continuous enrollment for medical and pharmacy benefit for ≥ 12 months before the index date (baseline) and ≥ 6 months after the index date (follow-up). Patients were excluded if there was any diagnosis of chronic thromboembolic pulmonary hypertension (CTEPH), LHD, or left-sided heart failure during the study period, any lung transplant surgery during the baseline or 6-month follow-up period, any PH diagnosis before the first observed COPD diagnosis, or diagnosis of right or unspecified heart failure during baseline (Fig. 1). A non-PH COPD cohort was similarly constructed to include patients without any PH diagnosis during the study period; pseudo-index dates were assigned to mimic the distribution of index dates in the PH-COPD cohort (Fig. 1).

### Study measures

Annualized all-cause, COPD-, and COPD/PH- related HCRU and associated costs were evaluated during the minimum 6-month follow-up period. HCRU evaluated included hospitalizations, emergency department [ED] visits, outpatient office visits, other medical services (laboratory and pathology test, radiology, surgery, medical procedures/supplies/products during office visits, other ancillary services), and prescription drugs filled. Risk of medical service encounters was evaluated by the number of patients with a specific event per 100 patient-years at risk. Time at risk referred to the time between the index date and the earliest date of the first event of interest, the end of continuous enrollment, or the end of the study period. Among patients with respective utilization, the number of encounters per patient per year (PPPY) were reported. COPD/PH-related medical events were identified by a primary diagnosis of COPD or PH on the claim. Utilization of any maintenance or rescue therapies for COPD was considered as COPD-related prescription drug utilization. The total costs associated with overall HCRU and each type of HCRU were measured by the contracted reimbursable amount of the covered care and reported in 2020 US dollars using the Consumer Price Index of Medical care for all Urban Consumers [12].

Patient demographics, comorbidities (including the Charlson Comorbidity Index that excluded respiratory conditions [non-respiratory CCI [13]] and the presence/absence of individual comorbid conditions), and COPD exacerbation history were assessed in the ≥ 12-month baseline period for the PH-COPD and non-PH COPD cohorts. The presence of a comorbidity/symptom was defined by ≥ 1 relevant diagnosis code. COPD exacerbations were identified by a claims-based algorithm where a severe exacerbation was indicated by a hospitalization with a primary diagnosis of COPD and a moderate exacerbation was indicated by an ED visit with a primary diagnosis of COPD or an outpatient visit with a COPD diagnosis accompanied by filling of a prescription for an oral corticosteroid (OCS) or antibiotic within 7 days of the visit [14].

### Statistical analysis

Patients in the PH-COPD cohort were 1:1 matched to patients in the non-PH COPD cohort based on propensity scores (PS) derived from patient demographic and baseline clinical characteristics, which accounted for the following: index year, age, gender, length of baseline period, US census region, payer type, plan type, non-respiratory CCI score, sleep apnea, atrial fibrillation, cardiovascular disease [CVD], diabetes, hypertension, heart failure, kidney disease, obesity, sinusitis, and any severe COPD exacerbation. Greedy nearest neighbor matching without replacement was performed using a caliper width of 0.01 of the standard deviation (SD) of the logit of the PS [15]. A standardized mean difference (SMD) of < 0.1 was used to indicate covariate balance between the PH-COPD and non-PH COPD cohorts before and after matching. Annualized all-cause HCRU and associated costs during the follow-up period were compared between the matched cohorts using the McNemar/Bowker test for categorical variables and paired t-test for continuous variables. A sensitivity analysis was conducted to compare all-cause HCRU between the subgroups of patients who received ≥ 1 COPD maintenance medication (i.e., monotherapy of long-acting beta agonist [LABA], long-acting muscarinic antagonist [LAMA], and inhaled corticosteroid [ICS]; dual therapy of ICS/LABA and LAMA/LABA; triple therapy of ICS/LAMA/LABA, as well as phosphodiesterase-4 inhibitors and Xanthines) during the study period in the matched PH-COPD and non-PH COPD cohorts and to describe COPD/PH-related HCRU in the subgroup of PH-COPD cohort. A generalized linear model (gamma distribution and log-link function) was used to identify patient characteristics associated with higher total all-cause healthcare costs in the unmatched PH-COPD cohort. For all analyses, a two-tailed test of significance was assumed, and the alpha level was set a-priori at 0.05. All data management and analyses were performed with SAS version 9.4 software (SAS Inc., Cary, North Carolina, USA).

## Results

### Description of the study cohorts

Of 273,792 patients with COPD identified, 2,224 and 83,667 patients were included in the PH-COPD cohort and non-PH COPD cohort, respectively (Table 1). Before matching, the PH-COPD and non-PH COPD cohorts were significantly different in most demographic and baseline clinical characteristics evaluated during the baseline period (median duration 28–29 months) (Table 1). Compared to the non-PH COPD cohort, the PH-COPD cohort was older (mean[± SD] age: 62.6[± 10.4] vs. 58.7[± 8.4] years), consisted of a higher proportion of females (61.3% vs. 57.3%;), patients covered by commercial Medicare (29.1% vs. 14.8%), and HMO enrollees (31.0% vs. 24.1%), and had a higher non-respiratory CCI score (mean[± SD]: 1.7[± 1.8] vs. 1.1[± 1.5]) as well as a higher proportion of patients with a CCI ≥ 3 (26.5% vs. 14.1%). Most respiratory comorbidities/symptoms (e.g., sleep apnea: 26.2% vs. 16.9%; dyspnea: 67.2% vs. 48.7%) and non-respiratory comorbidities (e.g., atrial fibrillation: 14.2% vs. 4.4%; CVD: 43.8% vs. 27.4%; obesity: 69.6% vs. 56.5%) also occurred at a higher frequency in the PH-COPD cohort compared to the non-PH COPD cohort. The presence of an associated connective tissue disease was rarely reported in both cohorts with a similar prevalence (< 2%).

**Table 1:** Patient demographic and baseline clinical characteristics before and after propensity score matching

Patient characteristics	Pre-matching (*N* = 85,901)			Post-matching (*N* = 4,448)		
	PH-COPD (*N* = 2,224)	Non-PH COPD (*N* = 83,677)	SMD	PH-COPD (*N* = 2,224)	Non-PH COPD (*N* = 2,224)	SMD
**Index year**						
2015	160 (7.2%)	3,996 (4.8%)	0.1202	160 (7.2%)	146 (6.6%)	0.0573
2016	739 (33.2%)	31,031 (37.1%)		739 (33.2%)	767 (34.5%)	
2017	584 (26.3%)	21,377 (25.5%)		584 (26.3%)	552 (24.8%)	
2018	431 (19.4%)	15,998 (19.1%)		431 (19.4%)	464 (20.9%)	
2019	310 (13.9%)	11,275 (13.5%)		310 (13.9%)	295 (13.3%)	
**Age at index**						
Mean (SD)	62.6 (10.4)	58.7 (8.4)	0.4035	62.6 (10.4)	62.4 (9.6)	0.0149
Median (IQR)	61.0 (56.0–66.0)	59.0 (54.0–63.0)		61.0 (56.0–66.0)	61.0 (57.0–65.0)	
Categories						
40–54 years	433 (19.5%)	23,970 (28.6%)	0.3397	433 (19.5%)	369 (16.6%)	0.0799
55–64 years	1,183 (53.2%)	47,349 (56.6%)		1,183 (53.2%)	1,252 (56.3%)	
65 + years	608 (27.3%)	12,358 (14.8%)		608 (27.3%)	603 (27.1%)	
**Female (%)**	1,363 (61.3%)	47,948 (57.3%)	0.0812	1,363 (61.3%)	1,335 (60.0%)	0.0258
**Census region**						
Northeast	408 (18.3%)	15,238 (18.2%)	0.1553	408 (18.3%)	408 (18.3%)	0.0623
Midwest	767 (34.5%)	25,905 (31.0%)		767 (34.5%)	708 (31.8%)	
South	756 (34.0%)	34,071 (40.7%)		756 (34.0%)	786 (35.3%)	
West	293 (13.2%)	8,463 (10.1%)		293 (13.2%)	322 (14.5%)	
**Payer type**						
Private insurance	904 (40.6%)	40,833 (48.8%)	0.3553	904 (40.6%)	907 (40.8%)	0.0437
Medicare	648 (29.1%)	12,407 (14.8%)		648 (29.1%)	610 (27.4%)	
Medicaid	117 (5.3%)	6,445 (7.7%)		117 (5.3%)	119 (5.4%)	
Other^a^	555 (25.0%)	23,992 (28.7%)		555 (25.0%)	588 (26.4%)	
**Plan type**						
HMO	689 (31.0%)	20,167 (24.1%)	0.1606	689 (31.0%)	657 (29.5%)	0.0344
PPO	1,426 (64.1%)	58,278 (69.6%)		1,426 (64.1%)	1,461 (65.7%)	
POS	69 (3.1%)	3,045 (3.6%)		69 (3.1%)	65 (2.9%)	
Other/unknown	40 (1.8%)	2,187 (2.6%)		40 (1.8%)	41 (1.8%)	
**Length of all-available baseline period**						
Mean (SD)	30.6 (13.1)	31.8 (13.0)	0.0919	30.6 (13.1)	30.6 (12.8)	0.0038
Median (IQR)	28.2 (19.3–39.6)	28.9 (21.0-41.1)		28.2 (19.3–39.6)	28.2 (19.7–40.2)	
**Non-respiratory CCI**^**b**^						
Mean (SD)	1.7 (1.8)	1.1 (1.5)	0.3616	1.7 (1.8)	1.7 (1.9)	0.0146
Median (IQR)	1.0 (0.0–3.0)	1.0 (0.0–2.0)		1.0 (0.0–3.0)	1.0 (0.0–3.0)	
Categories						
0	745 (33.5%)	40,300 (48.2%)	0.3017	745 (33.5%)	732 (32.9%)	0.0124
1	538 (24.2%)	20,327 (24.3%)	0.0024	538 (24.2%)	538 (24.2%)	0.0000
2	351 (15.8%)	11,254 (13.4%)	0.0661	351 (15.8%)	355 (16.0%)	0.0049
≥ 3	590 (26.5%)	11,796 (14.1%)	0.3128	590 (26.5%)	599 (26.9%)	0.0091
**Comorbidities/symptoms of interest**						
*Respiratory*						
Asthma	729 (32.8%)	28,281 (33.8%)	0.0216	729 (32.8%)	767 (34.5%)	0.0362
Allergic rhinitis or atopy	406 (18.3%)	20,445 (24.4%)	0.1512	406 (18.3%)	482 (21.7%)	0.0856
Dyspnea	1,495 (67.2%)	40,709 (48.7%)	0.3830	1,495 (67.2%)	1,492 (67.1%)	0.0029
Pneumonia/acute bronchitis/bronchiolitis	1,193 (53.6%)	45,701 (54.6%)	0.0196	1,193 (53.6%)	1,211 (54.5%)	0.0162
Sinusitis	616 (27.7%)	30,756 (36.8%)	0.1947	616 (27.7%)	583 (26.2%)	0.0334
Sleep apnea	582 (26.2%)	14,149 (16.9%)	0.2267	582 (26.2%)	610 (27.4%)	0.0284
*Non -respiratory*						
Anxiety/depression	906 (40.7%)	35,886 (42.9%)	0.0436	906 (40.7%)	1,019 (45.8%)	0.1027
Atrial fibrillation	316 (14.2%)	3,690 (4.4%)	0.3421	316 (14.2%)	328 (14.7%)	0.0153
Cardiovascular disease	975 (43.8%)	22,960 (27.4%)	0.3476	975 (43.8%)	959 (43.1%)	0.0145
Connective tissue disease	42 (1.9%)	1,209 (1.4%)	0.0347	42 (1.9%)	42 (1.9%)	0.0000
Diabetes	705 (31.7%)	20,377 (24.4%)	0.1642	705 (31.7%)	729 (32.8%)	0.0231
Dyslipidemia	1,434 (64.5%)	53,286 (63.7%)	0.0166	1,434 (64.5%)	1,581 (71.1%)	0.1418
Hypertension	1,706 (76.7%)	54,922 (65.6%)	0.2463	1,706 (76.7%)	1,706 (76.7%)	0.0000
Heart failure	236 (10.6%)	3,352 (4.0%)	0.2559	236 (10.6%)	233 (10.5%)	0.0044
Kidney disease	389 (17.5%)	7,136 (8.5%)	0.2688	389 (17.5%)	393 (17.7%)	0.0047
Liver disease	305 (13.7%)	10,021 (12.0%)	0.0520	305 (13.7%)	316 (14.2%)	0.0143
Osteoporosis	254 (11.4%)	6,539 (7.8%)	0.1226	254 (11.4%)	257 (11.6%)	0.0042
Systemic autoimmune disease^c^	186 (8.4%)	5,516 (6.6%)	0.0674	186 (8.4%)	171 (7.7%)	0.0248
**Non-missing BMI**^**d**^	779 (35.0%)	25,692 (30.7%)	0.0921	779 (35.0%)	861 (38.7%)	0.0765
Underweight/normal weight	132 (5.9%)	5,108 (6.1%)	0.0758	132 (16.9%)	127 (14.8%)	0.0601
Overweight	105 (4.7%)	6,066 (7.2%)	0.2629	105 (13.5%)	149 (17.3%)	0.1062
Obese	542 (24.4%)	14,518 (17.4%)	0.2733	542 (69.6%)	585 (67.9%)	0.0352
**With smoking cessation therapies**^**e**^	208 (9.4%)	9,917 (11.9%)	0.0812	208 (9.4%)	218 (9.8%)	0.0153
**Total all-cause healthcare costs PPPM (2020 USD)**^**e**^						
Mean (SD)	$1073.9 ($2158.0)	$649.2 ($1284.1)	0.2392	$1073.9 ($2158.0)	$891.7 ($1559.1)	0.0968
Median (IQR)	$456.5 ($174.6-$1142.0)	$265.6 ($103.8-$674.4)		$456.5 ($174.6-$1142.0)	$400.6 ($158.0-$958.5)	
**Number of COPD exacerbations PPPY**						
Any moderate or severe exacerbation^f^	758 (34.1%)	26,930 (32.2%)	0.0404	758 (34.1%)	755 (33.9%)	0.0029
Mean (SD)	1.4 (1.8)	0.9 (1.1)	0.3143	1.4 (1.8)	1.1 (1.7)	0.1170
Median (IQR)	0.7 (0.4–1.5)	0.6 (0.4–0.9)		0.7 (0.4–1.5)	0.6 (0.4–1.3)	
Any moderate exacerbation^f^	707 (31.8%)	25,851 (30.9%)	0.0193	707 (31.8%)	688 (30.9%)	0.0184
Mean (SD)	1.3 (1.6)	0.9 (1.0)	0.2984	1.3 (1.6)	1.1 (1.6)	0.0961
Median (IQR)	0.7 (0.4–1.5)	0.6 (0.4–0.9)		0.7 (0.4–1.5)	0.6 (0.4–1.2)	
Any severe exacerbation^f^	199 (8.9%)	3,063 (3.7%)	0.2189	199 (8.9%)	177 (8.0%)	0.0356
Mean (SD)	0.6 (0.5)	0.5 (0.5)	0.2222	0.6 (0.5)	0.6 (0.4)	0.1551
Median (IQR)	0.4 (0.3–0.7)	0.4 (0.3–0.6)		0.4 (0.3–0.7)	0.5 (0.3–0.7)	

*BMI *Body mass index, *CCI *Charlson Comorbidity Index, *HMO *Health maintenance organization, *IQR *Interquartile range, *PPO *Preferred provider organization, *POS *Point-of-service, *PPPM/Y *Per patient per month/year, *SD *Standard deviation, *SMD *Standardized mean difference

^a^Other payers included self-pay insurance and unknown payer type;

^b^CCI was calculated using Quan’s adaptation, excluding chronic pulmonary disease;

^c^Autoimmune diseases included graft-versus-host disease, inflammatory bowel disease, rheumatoid arthritis, scleroderma, Sjogren’s disease, and valvular heart disease;

^d^BMI was identified by ICD-9 or ICD-10 diagnosis codes and were expected to be underreported;

^e^Smoking cessation therapy and baseline total healthcare costs were examined in the 12-month baseline period;

^f^A moderate exacerbation was defined as an ED visit with a primary diagnosis of COPD, or an outpatient visit with a diagnosis of COPD accompanied with an oral corticosteroid or antibiotic within ± 7 days of the visit; a severe exacerbation was defined as a hospitalization with a primary diagnosis of COPD.

Before matching, the PH-COPD cohort had higher all-cause baseline healthcare costs ($1,073 vs. $649 per patient per month [PPPM]) and more patients with a history of severe COPD exacerbation in the 12-month baseline period (8.9% vs. 3.7%). All characteristics noted above had a SMD > 0.1.

All patients in the PH-COPD cohort were then matched 1:1 to patients in the non-PH COPD cohort (*N* = 2,224). After this, the two cohorts were well-balanced on all demographic characteristics and most clinical characteristics, with some residual difference (SMD > 0.1) observed in the baseline prevalence of dyslipidemia (SMD = 0.16; Table 1). All main analyses reported are based on this final matched population (*n* = 4,488). A total of 1,694 patients who received ≥ 1 COPD maintenance medication during the study period were identified from the matched population and included in the sensitivity analysis.

### Follow-up HCRU

**Fig. 2:**
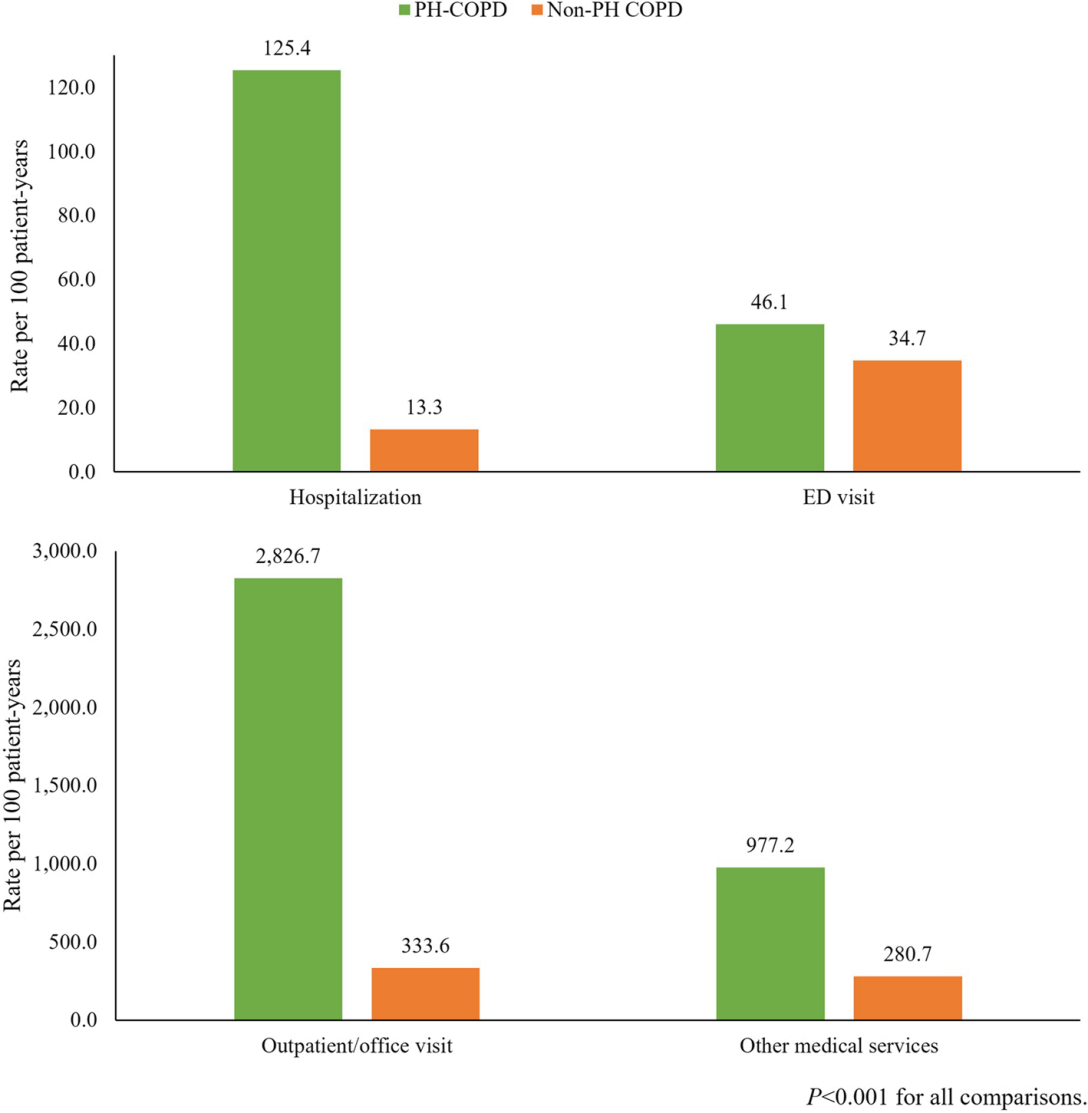
All-cause medical service utilization during the follow-up period*. *ED, emergency department. Other medical services included laboratory and pathology test, radiology, surgery, medical procedures/supplies/products during office visits, and other ancillary services

All-cause utilization rates were higher in the PH-COPD cohort compared to the matched non-PH COPD cohort across all medical service categories, with the largest difference observed for hospitalizations (Fig. 2). The risk of hospitalization was about 9 times higher in the PH-COPD cohort vs. the non-PH COPD cohort (125.4 vs. 13.3 per 100 patient-years or 63 vs. 7 out of 100 patients with a hospitalization within 6 months; *p* < 0.001). Furthermore, among patients with ≥ 1 hospitalization, the median (interquartile range [IQR]) number of hospitalizations (1.1 [0.6–1.9] vs. 0.7 [0.4–1.3] PPPY, *p* < 0.001) and length of stay (5.7 [2.8–12.2] vs. 3.1 [1.5–7.7] days PPPY, *p* < 0.001) were also higher in the PH-COPD cohort. Similar findings of all-cause HCRU were observed in the sensitivity analysis that compared subgroups of the PH-COPD and non-PH COPD matched cohorts who received ≥ 1 maintenance COPD treatment during the study period (Supplemental Fig. 1).

**Fig. 3:**
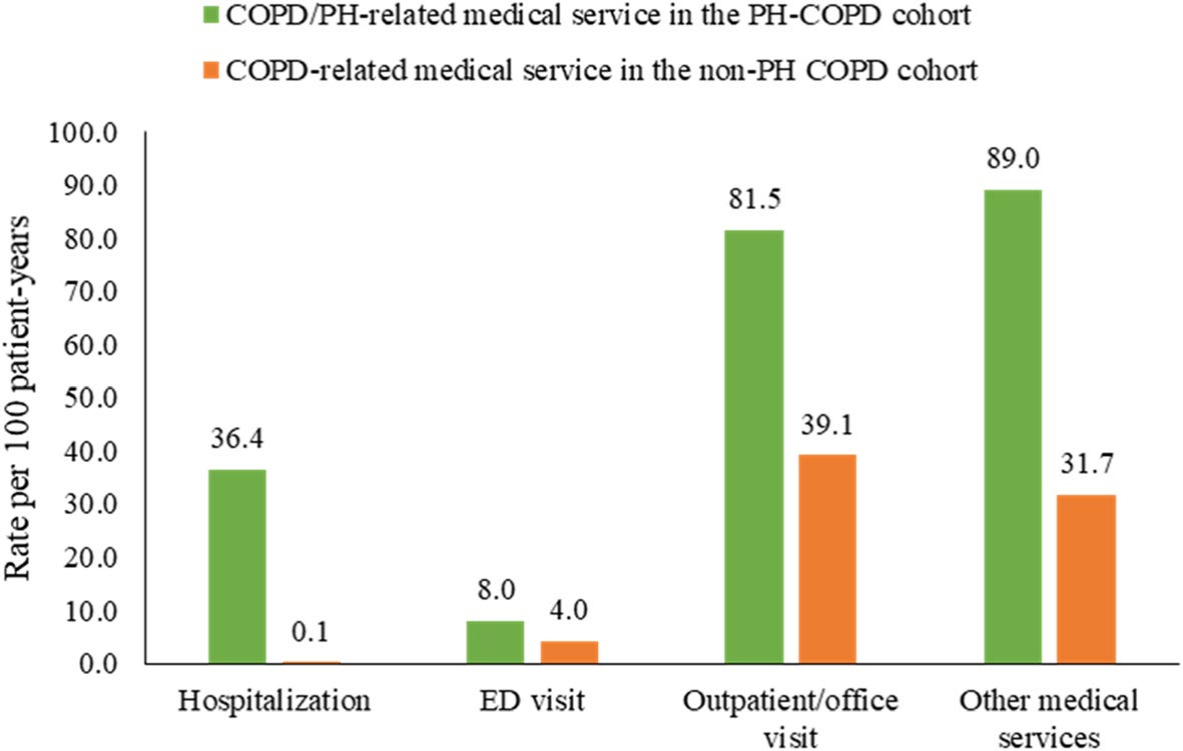
COPD/PH-related and COPD-related medical service utilization during the follow-up period*. *ED, emergency department. Other medical services included laboratory and pathology test, radiology, surgery, medical procedures/supplies/products during office visits, and other ancillary services. COPD-related medical services (hospitalization, ED visit, outpatient/office visit, other medical service) were identified by a primary diagnosis of COPD on claims. COPD/PH-related medical services (hospitalization, ED visit, outpatient/office visit, other medical service) were identified by a primary diagnosis of COPD or PH on claims. Noting that COPD/PH-related medical service utilization in the PH-COPD cohort was not statistically compared to COPD-related utilization in the non-PH COPD cohort

**Fig. 4:**
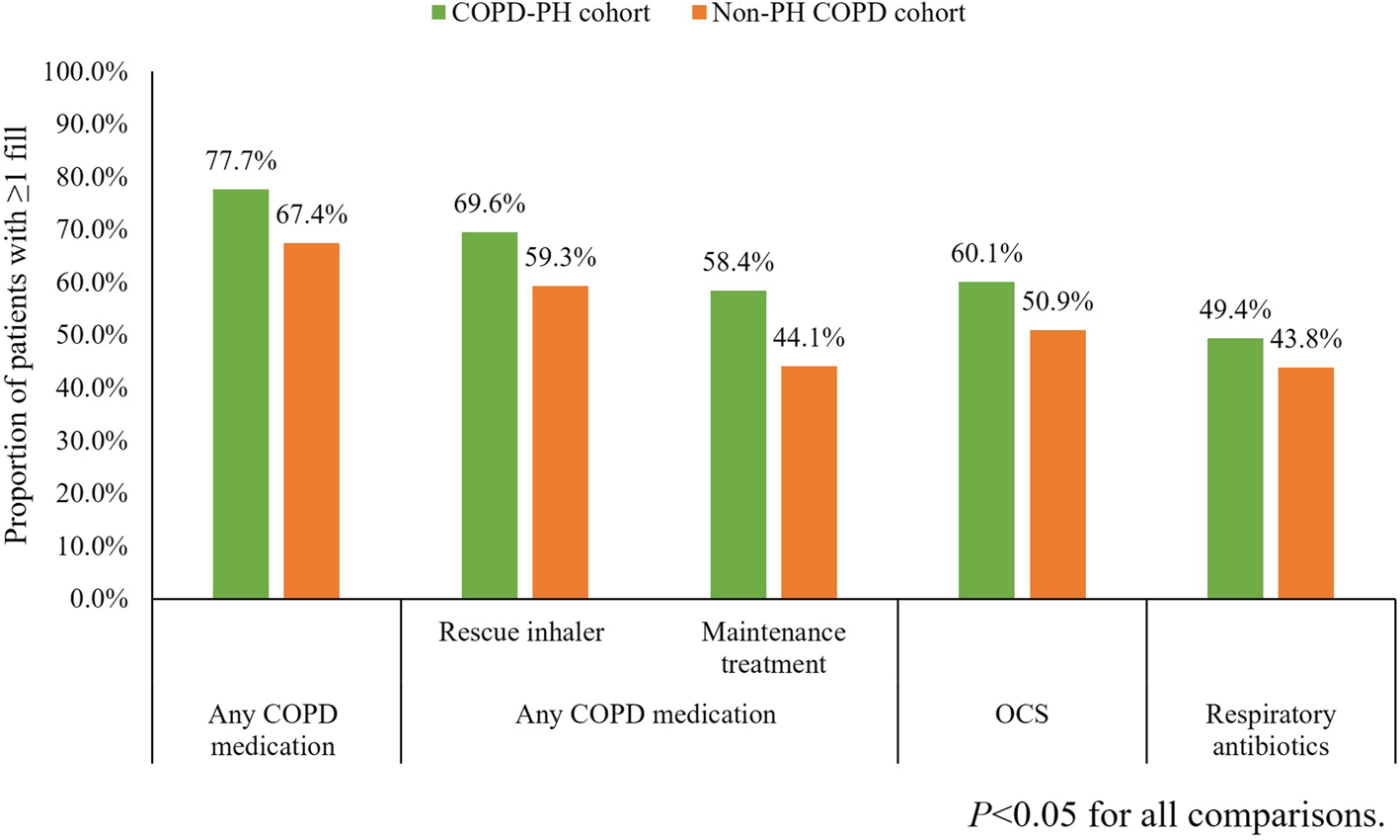
COPD-related pharmacy utilization during the follow-up period*. *OCS, oral corticosteroids. Maintenance treatment included long-acting beta_2_ agonists (LABA), long-acting muscarinic antagonists (LAMA), LABA/LAMA, inhaled corticosteroids (ICS), LABA/ICS, fixed dose triple therapy of ICS/LAMA/LABA, Phosphodiesterase-4 inhibitors, and xanthines. Rescue inhalers included short-acting beta_2_ agonists (SABA), short-acting muscarinic antagonists (SAMA), SABA/SAMA

In the PH-COPD cohort, 36.4 out of 100 patients had a COPD/PH-related hospitalization within a year after the PH diagnosis (Fig. 3). Among patients with ≥ 1 COPD/PH-related hospitalization(s) during follow-up, the cohort had a median (IQR) number of 0.8 (0.5–1.5) hospitalizations and median (IQR) stay of 2.6 (1.1–6.4) days PPPY. In the non-PH COPD cohort, only 1 out of 1,000 patients had a COPD-related hospitalization within a year after the COPD diagnosis. There were 82 out of 100 patients having a COPD/PH-related outpatient/office visit and 89 out of 100 patients having a COPD/PH-related other medical service visit within a year of follow up in the PH-COPD cohort, whereas COPD-related outpatient/office (39 per 100 patient-years) and other medical services visits (32 per 100 patient-years) were less frequently observed in the non-PH COPD cohort. Less than 10 out of 100 patients in the PH-COPD and non-PH COPD cohort had a COPD/PH-related or COPD-related ED visit within a year of follow-up (Fig. 3). Similar findings of COPD/PH-related medical service usage were observed in the sensitivity analysis (Supplemental Fig. 2). In terms of pharmacy utilization, the usage of any COPD maintenance treatment (58.4% vs. 44.1%), rescue inhaler (69.6% vs. 59.3%), OCS (60.1% vs. 50.9%), and antibiotic (49.4% vs. 43.8%) was more frequently observed in the PH-COPD cohort compared to the non-PH COPD cohort (Fig. 4).

### Follow-up healthcare costs

**Fig. 5:**
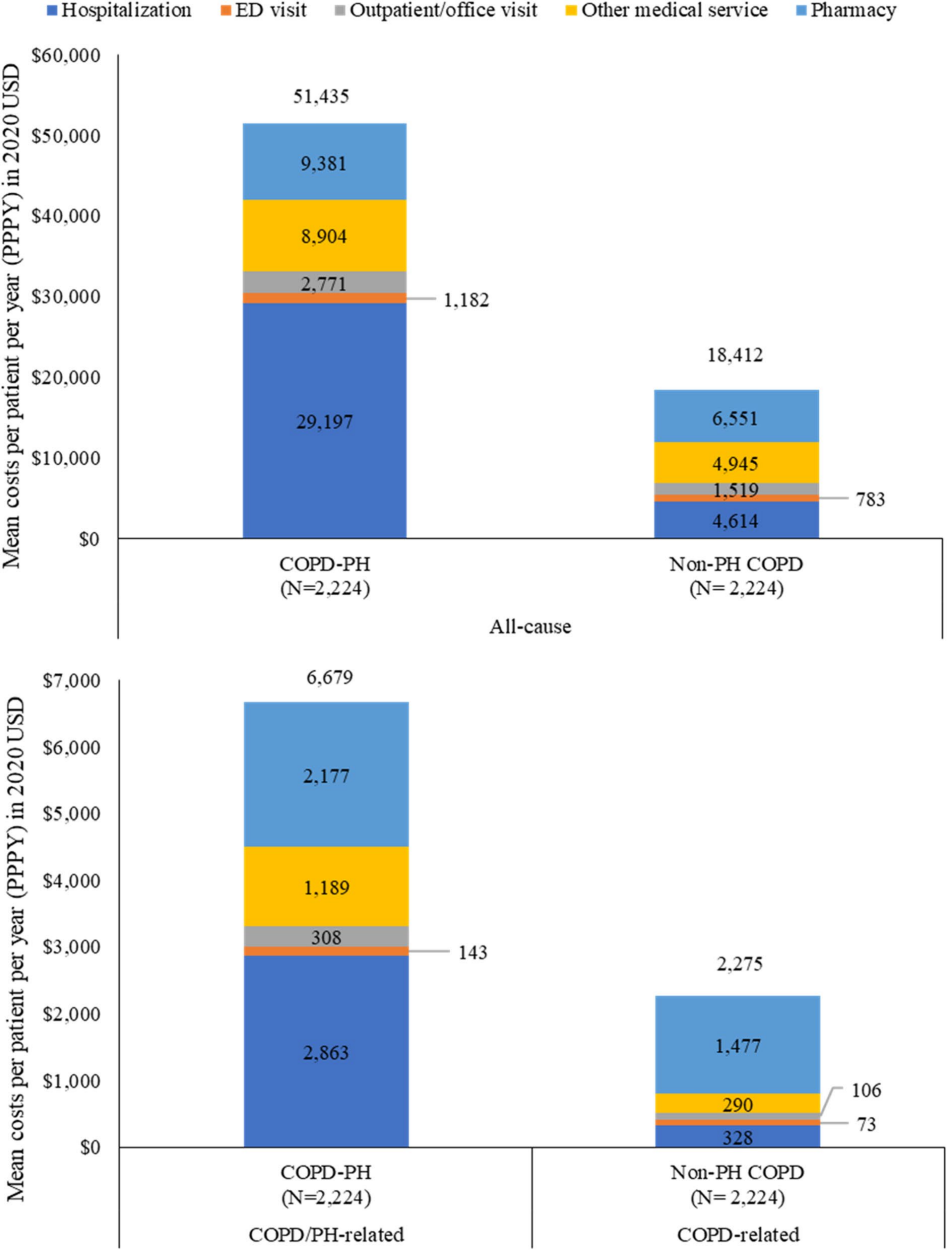
Healthcare costs during follow-up. ED, emergency department. COPD-related medical costs (hospitalization, ED visit, outpatient/office visit, other medical service) were identified by a primary diagnosis of COPD on claims. COPD/PH-related medical costs (hospitalization, ED visit, outpatient/office visit, other medical service) were identified by a primary diagnosis of COPD or PH on claims. All costs were estimated as contracted reimbursable amount of the covered care

The PH-COPD cohort had higher all-cause healthcare costs PPPY compared to the matched non-PH COPD cohort, overall and across all service categories (Fig. 5). The mean (± SD) total all-cause healthcare costs PPPY in the PH-COPD cohort were $51,435 (±$62,631) during follow-up. This was almost three times higher than those of the matched non-PH COPD cohort ($18,412 [±$27,323], *p* < 0.001). Hospitalization was the main driver of total healthcare costs in the PH-COPD cohort, accounting for 56.8% of the total all-cause costs. Pharmacy utilization was the major cost driver in the non-PH COPD cohort, contributing to 35.6% of the total all-cause costs.

The PH-COPD cohort had a mean of $6,679 (±$13,585) for COPD/PH-related medical costs PPPY, representing 13.0% of the all-cause costs. Specifically, the COPD/PH-related medical costs and COPD-related pharmacy costs were $4,502 (±$12,578) and $2,177 (±$2,986) PPPY, accounting for 10.7% and 23.2% of all-cause medical and pharmacy costs, respectively. The non-PH COPD cohort had a mean of $2,275 (± 4,531) for COPD-related healthcare costs PPPY, representing 12.4% of the all-cause costs. COPD-related pharmacy costs accounted for 65.0% of the total COPD-related costs (Fig. 5).

In the PH-COPD cohort, baseline patient characteristics that were significantly associated with higher total all-cause costs included being male (vs. female), residing in the West or Midwest US (vs. the Northeast), having a higher CCI score, having anxiety/depression or systemic autoimmune disease, and having ≥ 1 prior moderate or severe COPD exacerbation (Table 2). The largest incremental costs were observed for having autoimmune disease ($9,706), followed by residing in the West ($8,274), and having two or more moderate/severe exacerbations during baseline ($5,216; all *p* < 0.01).

**Table 2:** Patient characteristics associated with total all-cause healthcare costs in the PH-COPD cohort

Patient baseline characteristics	Incremental $	Beta^a^	95% CI	*P* value^a^
**Intercept**	**$41,274**	10.628	(10.411, 10.845)	< 0.0001
**Index year**				
2015 (Reference)				
2016	$0	0.000	(-0.152, 0.152)	0.9999
2017	-$3,219	-0.081	(-0.238, 0.075)	0.3096
2018	-$5,238	-0.136	(-0.297, 0.025)	0.0981
2019	$7,317	0.163	(-0.008, 0.334)	0.0610
**Age group, years**				
40–54 (Reference)				
55–64	$1,342	0.032	(-0.068, 0.132)	0.5307
65+	-$5,944	-0.156	(-0.328, 0.017)	0.0766
**Sex**				
Female (Reference)				
Male	$4,638	0.107	(0.027, 0.186)	0.0088
**Census region**				
Northeast (Reference)				
Midwest	$3,304	0.077	(-0.031, 0.185)	0.1631
South	$415	0.010	(-0.101, 0.121)	0.8597
West	$8,274	0.183	(0.048, 0.317)	0.0078
**Payer type**				
Private insurance (Reference)				
Medicare	-$17,125	-0.536	(-0.708, -0.364)	< 0.0001
Medicaid	-$19,133	-0.623	(-0.822, -0.424)	< 0.0001
Other^b^	$1,965	0.047	(-0.048, 0.141)	0.3364
**Plan type**				
HMO (Reference)				
PPO	-$3,040	-0.077	(-0.182, 0.029)	0.1565
Other^c^	-$3,116	-0.079	(-0.274, 0.117)	0.4320
**Non-respiratory CCI score**	$5,272	0.120	(0.096, 0.144)	< 0.0001
**Comorbidities (yes vs. no)**				
Asthma	$1,138	0.027	(-0.058, 0.112)	0.5303
Pneumonia or acute bronchitis/bronchiolitis	$5,669	0.129	(0.047, 0.211)	0.0021
Sinusitis	-$4,729	-0.122	(-0.208, -0.035)	0.0058
Sleep apnea	-$4,568	-0.117	(-0.207, -0.028)	0.0101
Anxiety/depression	$5,477	0.125	(0.045, 0.204)	0.0020
Cardiovascular disease	-$288	-0.007	(-0.093, 0.079)	0.8726
Dyslipidemia	-$4,040	-0.103	(-0.191, -0.015)	0.0220
Hypertension	$4,318	0.100	(0.002, 0.197)	0.0459
Systemic autoimmune disease	$9,706	0.211	(0.074, 0.349)	0.0026
**Number of prior moderate or severe exacerbations**				
0 (reference)				
1	$3,397	0.079	(-0.027, 0.186)	0.1453
2+	$5,216	0.119	(0.016, 0.222)	0.0234

*CCI *Charlson Comorbidity Index, *HMO *Health maintenance organization, *PPO *Preferred provider organization, *PPPY *Per patient per year, *SE *Standard error, *95%CI *95% Confidence interval

^a^Parameters were estimated from generalized linear model of total all-cause healthcare costs PPPY during follow-up, with log-link and gamma distribution;

^b^Other payers included self-pay insurance and unknown payer type;

^c^Other plans included point-of-service, consumer-directed and indemnity plan

## Discussion

To our knowledge, this is the first real-world study to estimate the direct healthcare costs associated with PH-COPD among patients with COPD and to identify patient characteristics that are associated with higher healthcare costs among these patients. A claims-based algorithm was employed to identify PH-COPD based on PH diagnosis while excluding patients with diagnoses of other possible causes of PH. Based on this algorithm, less than 1% of patients with COPD were identified as having PH-COPD in this study. The prevalence of PH in COPD patients has been reported ranging from 5–53% [5]. The relative low prevalence of PH-COPD is possibly an underestimate since the diagnosis of PH-COPD is rare. This may, in part, be because there are no guidelines that recommend screening for PH-COPD or performing confirmatory RHC in patients with COPD [6, 7]. It is also plausible that COPD patients with PH are assigned codes associated with other PH groups in order to enable prescribing of PAH treatment(s) or some patients with PH-COPD who had a COPD diagnosis prior to the start of the study period (October 1, 2014) were excluded during the patient selection process.

Consistent with a previous retrospective study on Group 3 PH and a study on acute exacerbations of COPD (AECOPD) [11, 16], our study observed a higher proportion of females and a higher comorbidity burden in patients with PH-COPD compared to COPD patients without PH. Although there is evidence indicating females are at a higher risk of PAH [17], the sex differences in other types of PH, including PH-COPD, are less well explored. There is growing evidence that female COPD patients have earlier disease onset, lower smoking exposure, and are more prone to lung function impairment as well as more severe disease [18]. However, studies examining the clinical implication of sex differences in PH-COPD are lacking. Patients with PH-COPD were also older than COPD patients without PH in our study. Elderly COPD patients may be more prone to the development of PH due to cardiopulmonary comorbidities, such as CVD, heart failure, thromboembolic disease and sleep apnea. It is therefore incumbent on clinicians to be mindful of and address these comorbidities in the “holistic” care of COPD patients.

Our findings suggest that PH-COPD is associated with a significant excess burden on the healthcare system. The total all-cause healthcare costs PPPY were almost three times greater in patients with PH-COPD as compared to COPD patients without PH ($51,435 vs. $18,432). This cost estimate is similar to a previous US database study estimating the cost burden for patients with Group 3 PH ($44,732). Hospitalizations were the primary driver of total costs of patients with PH-COPD in our study and the study of Group 3 PH [16]. The higher hospitalization costs in patients with PH-COPD from more frequent hospitalizations and longer hospital stays are likely due to the added care burden imposed by complicating PH. Munshi et al. reported that the presence of PH was associated with a higher likelihood of having 30-day re-admission, > 3 days of hospitalization, and an invasive procedure, such as a tracheostomy, bronchoscopy, or chest tube placement among patients with AECOPD [11]. Preventing hospitalization through optimal management of COPD may help reduce the burden of PH-COPD. However, only 58% of patients identified as having PH-COPD received ≥ 1 COPD maintenance medication after the first observed PH diagnosis during follow-up in our study, despite their known diagnosis of COPD. Future analysis comparing patients with PH-COPD receiving COPD maintenance treatment vs. those who are not may help identify characteristics of patients who are undertreated and associated barriers that could be addressed. As the use of COPD maintenance treatment and treatment adherence can also reflect disease severity of COPD, further analysis exploring the association between adherence to COPD maintenance treatment and the burden of PH-COPD is warranted. As the recommended care for patients with PH-COPD is to optimally treat the underlying COPD [6], it is also possible that patients in the PH-COPD cohort who did not have any COPD maintenance treatment during the study period included some patients with other types of PH. However, our sensitivity analysis among patients with PH-COPD and COPD patients without PH who received ≥ 1 COPD maintenance medication demonstrated similar all-cause and COPD/PH-related HCRU.

Our findings suggest that having systemic autoimmune disease, living in the West or Midwest US, and having two or more prior moderate or severe COPD exacerbations, are major risk factors for higher total costs among patients with PH-COPD. It is well established that systemic autoimmune diseases such as rheumatoid arthritis are associated with a substantial cost burden. It is also plausible that some PAH patients with COPD as a comorbidity were inadvertently misclassified as PH-COPD patients. Indeed, this is a common conundrum, and in fact about 14% of patients from the REVEAL Registry of PAH patients had comorbid COPD [19]. The geographic variation of PH-COPD burden observed in this study may be related to multiple factors, including but not limited to access to care and race/ethnicity, which were not captured by the study data. Further studies, including such residual confounders, are needed to better identify high-risk populations. Lastly, Anzueto et al. reported COPD exacerbations as a major driver of the burden of COPD, accounting for over $18 billion of direct medical costs annually [20]. Since the presence of PH is associated with an increased risk of COPD exacerbations [21] and having prior exacerbations is a large cost driver among PH-COPD patients in this study, there appears to be an unmet need for well-designed randomized controlled studies evaluating treatments targeting PH. Indeed, the prevention of COPD acute exacerbations should be evaluated in such future clinical trials.

Several limitations of the study should be noted. First, there is no specific diagnosis code for PH-COPD. Patients with PH-COPD were identified by a claims-based algorithm, which may have led to potential misclassification of PAH or PH-LHD as PH-COPD in the study. Specifically, the lack of lung function, hemodynamic, and echocardiogram data in administrative claims, limited our ability to better categorize these patients or examine a specific patient subgroup (e.g., patients with pulmonary artery pressure > 35 mmHg). The prevalence of PH was likely underestimated as only 1% of patients with COPD were identified having PH based on the algorithm. It is therefore likely that a sizeable number of patients with PH-COPD were misclassified into the non-PH COPD cohort, thereby potentially narrowing the differences noted between the two groups. A future study leveraging natural language processing with clinical note data may help improve identification of patients with PH-COPD and enable further study of PH-COPD subpopulations or phenotypes and capture patient characteristics (e.g. GOLD stage, lung function, and smoking status and history) that could influence study findings. Further, costs were estimated from the third-party paid amount for covered care in administrative claims; therefore, the costs of clinically relevant events that do not require health care services, such as mild exacerbations, and services that are not documented in claims data (e.g., medications not covered by insurance or those obtained through cash payments) were not captured in this study. Thus, the direct healthcare costs associated with PH-COPD could have been underestimated. In addition, costs attributable to COPD or PH were identified by primary diagnosis codes recorded on claims and may have been underestimated due to not accounting for the coding of other comorbidities being prioritized and off-label use of treatments (e.g., PAH medications). Although PH-COPD is associated with substantially higher costs, only a small proportion (13%) of the total healthcare costs of patients with PH-COPD were primarily attributable to COPD or PH; however, this is likely an underestimation resulting from only attributing costs based on the primary diagnosis codes in administrative claims. About half of the study cohorts had two months of follow-up (March 1 through May 31, 2020) during the early COVID-19 pandemic period. Although COVID-19 related service use, defined by ICD-10-CM diagnosis codes of B34.2, B97.2, U07.1, and U07.2, was rarely (0.2–0.3%) observed in the study cohorts, HCRU and associated costs during this period may be different from those prior to the pandemic. Future studies exploring the impact of COVID-19 on patients with COPD and PH-COPD are warranted. Lastly, the study results were based on data from a younger, commercially insured population and may not be generalizable to the overall US COPD population.

## Conclusions

This study employed a claims-based algorithm to identify patients with PH-COPD. PH-COPD presents a substantial economic burden among US patients with COPD, especially among patients with frequent severe exacerbations and a high comorbidity burden. The additional costs accrued by PH-COPD patients appeared largely attributable to comorbidities and hospitalizations in this population. Future studies examining costs associated with different comorbidities among patients with PH-COPD may help better understand the burden of PH-COPD and the patient populations to target to reduce the burden. This analysis may help lay the foundation and provides further impetus for randomized controlled trials in PH-COPD to best identify the COPD phenotype(s) who might benefit from PH-specific therapies [22, 23].

### Supplementary Information

**Additional file 1.**

**Additional file 2.**

**Additional file 3.**

## Data Availability

The data that support the findings of this study are available from IQVIA, but restrictions apply to the availability of these data, which were used under license for the current study, and so are not publicly available. Data are however available from the corresponding authors upon reasonable request and with permission of IQVIA.
