# Supplementary material for: Healthcare resource utilization in patients with pulmonary hypertension associated with chronic obstructive pulmonary disease (PH-COPD): a real-world data analysis
Source: BMC Pulm Med. 2023 Nov 21;23:455. doi: 10.1186/s12890-023-02698-9 (PMC10664271; doi:10.1186/s12890-023-02698-9)

**Supplemental Figure 2. COPD/PH-related medical service utilization during the follow-up period of PH-COPD subgroup who received ≥1 maintenance treatment during the study period**

ED, emergency department.

Other medical services included laboratory and pathology test, radiology, surgery, medical procedures/supplies/products during office visits, and other ancillary services.


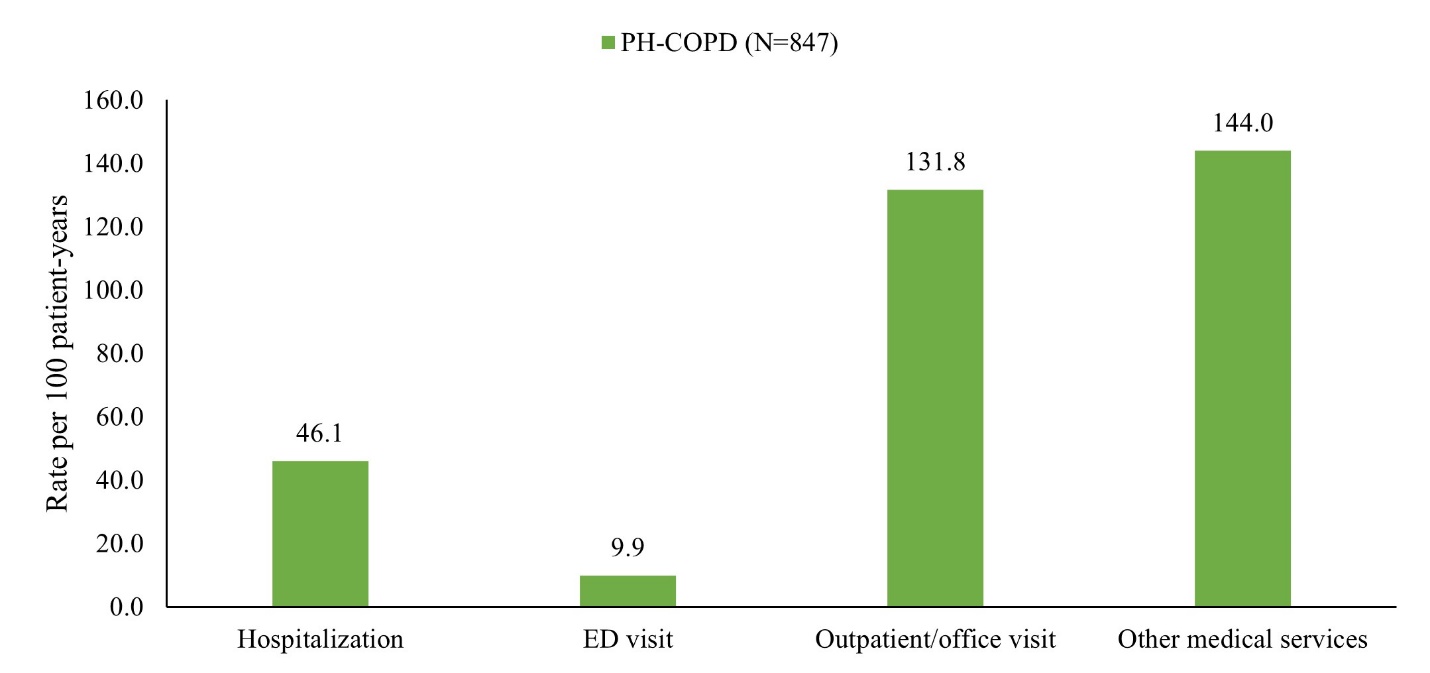

Supplement: Supplementary file 2 — Additional file 2. [file 12890_2023_2698_MOESM2_ESM.docx]
