# Supplementary material for: Healthcare resource utilization in patients with pulmonary hypertension associated with chronic obstructive pulmonary disease (PH-COPD): a real-world data analysis
Source: BMC Pulm Med. 2023 Nov 21;23:455. doi: 10.1186/s12890-023-02698-9 (PMC10664271; doi:10.1186/s12890-023-02698-9)
Supplement: Supplementary file 3 — Additional file 3. [file 12890_2023_2698_MOESM3_ESM.docx]

**Supplemental Figure 1. All-cause medical service utilization during the follow-up period of subgroups who received ≥1 maintenance treatment during the study period**

ED, emergency department.

Other medical services included laboratory and pathology test, radiology, surgery, medical procedures/supplies/products during office visits, and other ancillary services.

Among patients with at least 1 hospitalization, patients had a median (IQR) stay of 5.7 (2.8-12.1) nights per patient per year for patients in the PH-COPD cohort who received ≥1 maintenance treatment during the study period and a median (IQR) stay of 3.2 (1.6-8.1) nights in the matched subgroup of the non-PH COPD cohort.

**Supplemental Figure 2. COPD/PH-related medical service utilization during the follow-up period of PH-COPD subgroup who received ≥1 maintenance treatment during the study period**

ED, emergency department.

Other medical services included laboratory and pathology test, radiology, surgery, medical procedures/supplies/products during office visits, and other ancillary services.
